# Severe Thrombocytopenia Secondary to Systemic Lupus Erythematosus With Antiphospholipid Antibodies in a Middle-Aged Woman

**DOI:** 10.7759/cureus.62804

**Published:** 2024-06-20

**Authors:** Yuki Matsuura, Tomoko Tomita, Makoto Kondo, Masaya Mukai, Hiroshi Kataoka

**Affiliations:** 1 Rheumatology and Clinical Immunology, Sapporo City General Hospital, Sapporo, JPN

**Keywords:** hydroxychloroquine treatment, oral glucocorticoids, immune thrombocytopenia purpura, systemic lupus erythematosus, antiphospholipid syndrome

## Abstract

Thrombocytopenia is a common hematological complication of systemic lupus erythematosus (SLE). However, severe thrombocytopenia is a relatively rare presentation, accounting for only 3-10% of cases. A 52-year-old woman was being treated with 4 mg/day of prednisolone for 12 years for SLE-induced autoimmune hemolytic anemia. She presented to her family physician with nasal bleeding and purpura, which required more than two hours to control. She had bruises on her legs and mild multiple arthralgia. The platelet count was 19,000/µL. She was suspected to have developed immune thrombocytopenia as an exacerbation of SLE. Thus, she was referred to our hospital. Laboratory examination revealed thrombocytopenia, hypocomplementemia, and a positive result for anti-cardiolipin (CL) and anti-β_2_-glycoprotein (GP) I IgG antibodies. The patient was diagnosed with thrombocytopenic purpura, complicated by SLE. Methylprednisolone pulse therapy, followed by 60 mg/day of prednisolone and 200/400 mg of hydroxychloroquine on alternate days, was initiated. The platelet count increased from 5,000/µl to 50,000/µl, and the immature platelet fraction (IPF) decreased from 14.9% to 6.3%. Anti-CL and anti-β_2_-GPI IgG antibodies were considered to be associated with thrombocytopenia and a risk of thrombotic events after normalization of her platelet counts. Therefore, aspirin therapy was initiated to prevent thrombosis. As an episode of acute thrombocytopenia occurred without other clinical findings indicating active SLE, it was important to determine the exact cause of thrombocytopenia in this situation. Immediate recovery of thrombocytopenia with high-dose prednisolone reduced the risk of bleeding that could have otherwise been fatal.

## Introduction

Thrombocytopenia can occur in various diseases. It is essential to take a detailed history of the present, past, and familial illnesses and social disorders, perform a thorough physical examination, and carry out laboratory tests to determine the underlying disease. Thrombocytopenia can occur due to autoimmunity, infections, hematologic disorders, and other conditions. Systemic lupus erythematosus (SLE) is an inflammatory disease with cutaneous, musculoskeletal, mucomembranous, neural, and hematological manifestations. Although thrombocytopenia is a common complication of SLE, severe thrombocytopenia (≤50,000/µL) is relatively rare, accounting for 3-10% of the cases [1,2]. Here, we report the case of a patient with low disease activity SLE with prednisolone-managed autoimmune hemolytic anemia (AIHA) who developed bleeding symptoms and severe thrombocytopenia without any trigger.

## Case presentation

A 52-year-old woman presented with complaints of mild arthralgia, features of subcutaneous hemorrhage, and nasal bleeding that required more than two hours to control. Twelve years ago, the patient was determined to have anemia during a gynecological surgery. As a result of the examination, SLE was diagnosed according to the 1997 American College of Rheumatology criteria (photosensitivity, hemolytic anemia, antinuclear antibodies, hypocomplementemia, neutropenia, and biologically false positive reactions) [2]. She had never experienced thrombocytopenia, the presence of schizocytes, renal dysfunction, or bleeding tendencies until this episode. Her AIHA was being treated with 40 mg/day of prednisolone, which was gradually reduced to 4 mg/day. None of her relatives had blood or immune disorders. She did not have any recent history of infection or vaccination. She had been on 4 mg/day of prednisolone and 40 mg of famotidine for a long period, which had not been modified until the recent bleeding episode. No new medications or heparin had been recently initiated. Physical examination revealed no abnormal findings, except for petechial hemorrhage on the dorsal surface of the chest and abdomen, and bruises on the arms and legs. The laboratory data (Table 1) were as follows: white blood cell count 5,600/μl, hemoglobin 12.3 g/dl, platelet count 5,000/µl, increased immature platelet fraction (IPF) 14.9% (normal: <6.1%), total bilirubin 2.9 mg/dl, haptoglobin 2.9 mg/dl, no lactate dehydrogenase (LDH) elevation (215 U/l), slightly decreased C_3_ (69.2 mg/dl) and CH_50_ (28.7 U/ml), and positive results for anti-cardiolipin and anti-β_2_ glycoprotein (GP)-I IgG antibodies.

**Table 1:** Laboratory data ALT: alanine aminotransferase; anti-β2 GPI: anti-β2 glycoprotein I antibodies; anti-CL: anti-cardiolipin antibodies; APTT: activated partial thromboplastin time; AST: aspartate aminotransferase; Cre: creatinine; CRP: C-reactive protein; D-Bil: direct bilirubin; eGFR: estimated glomerular filtration rate; Hb: hemoglobin; Hct: hematocrit; IPF: immature platelet fraction; LDH: lactate dehydrogenase; Plt: platelet; PT-INR: prothrombin time-international normalized ratio; RBC: red blood cell; Ret: reticulocyte; sIL-2R: soluble interleukin-2 receptor; T-Bil: total bilirubin; UN: urea nitrogen; WBC: white blood cell

Parameters	Patient value	Reference range
WBC	5,600	3,300-8,600/µl
RBC	4.26 × 10^6^	435-555/µl
Hb	12.3	13.7-16.8 g/dl
Hct	35.9	40.7-50.1%
Ret	2.2	0.5-2.5%
Plt	5,000	158-348 × 10^3^/µl
IPF	14.9	0.0-6.1%
PT-INR	0.97	0.8-1.2
APTT	31	25-35 seconds
Fibrinogen	291	200-400 mg/dl
D-dimer	<0.5	<0.5 µg/ml
T-Bil	2.9	0.4-1.5 mg/dl
D-Bil	0.1	0.0-0.2 mg/dl
AST	17	13-30 U/l
ALT	13	10-42 U/l
LDH	215	124-222 U/l
UN	13.6	8-20 mg/dl
Cre	0.8	0.65-1.07 mg/dl
eGFR	58.9	ml/min/1.73 m^2^
Haptoglobin	2.9	19-170 mg/dl
Ferritin	95	13-250 ng/ml
CRP	0.05	0.00-0.14 mg/dl
C_3_	69.2	72-135 mg/dl
C_4_	11.6	9.9-31.5 mg/dl
CH_50_	28.7	31.6-57.6 U/ml
sIL-2R	355	122-496 U/ml
Rheumatoid factor	<4	0-15 IU/ml
Antinuclear antibody	Homogeneous 1:40	
Anti-DNA antibody	<2.0	≤6.0 IU/ml
Anti-CL IgG antibody	53.2	<20 U/ml
Anti-β_2_GPI IgG antibody	606.1	<20 U/ml
Direct Coombs test	3+	

The coagulation-fibrinolytic system tests were normal. HAS-BLED score was 0 [3]. Urinalysis did not reveal any abnormal findings or sedimentation. Whole-body computed tomography revealed no specific findings, including splenomegaly.

**Figure 1:**
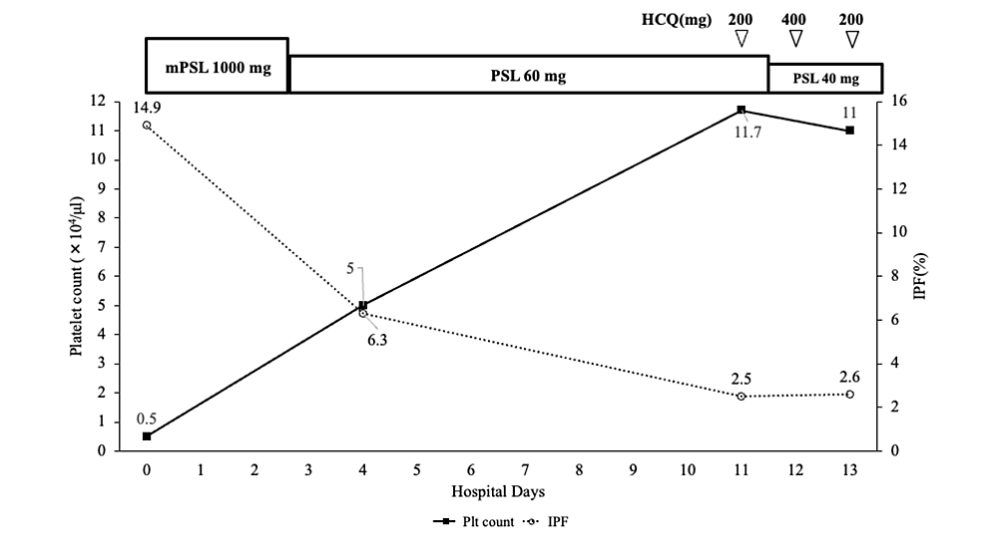
Patient’s in-hospital course HCQ: hydroxychloroquine; IPF: immature platelet fraction; mPSL: methylprednisolone; PSL: prednisolone

The patient’s in-hospital course is shown in Figure 1.

The patient was thought to develop secondary Evans syndrome based on mild hemolysis and possibly life-threatening thrombocytopenia with purpura related to SLE and received methylprednisolone pulse therapy (1,000 mg/day) from the day of admission for two days [4]. Thereafter, 60 mg/day of prednisolone was initiated. Upper gastrointestinal endoscopy was performed on the seventh day due to the detection of anti-*Helicobacter pylori* IgG antibodies. The patient was diagnosed with closed type-3 atrophic gastritis. The stool analysis was negative for *H. pylori *antigens, suggesting a past infection. Therefore, SLE was considered the cause of thrombocytopenia, and 200/400 mg of hydroxychloroquine (HCQ) every other day was initiated. Aspirin (100 mg/day) was concomitantly used to prevent spontaneous arterial or venous thrombosis due to the rapid recovery of platelet counts. On the 12th day, the prednisolone dose was reduced to 40 mg/day. Thirteen days after admission, the patient’s platelet count had recovered to 110,000/μl, and she was discharged from the hospital with a stable platelet count. Six months later, the platelet count was 157,000/μl, although complements were still slightly decreased (C_3_ 65.5 mg/dl, CH_50 _28.6U/ml), and anti-CL, PA-IgG (108 ng/10^7^ cells (normal upper limit: 46)), and anti-β_2_ GPI IgG antibodies were still positive while treated with 9 mg/day of prednisolone.

## Discussion

Thrombocytopenia is defined as a platelet count <150,000/μl [5]. It is classified as follows according to the degree of severity: mild (100,000-150,000/μl), moderate (50,000-100,000/μl), and severe (≤50,000/μl). It manifests as mucosal and subcutaneous bleeding. Severe thrombocytopenia can be fatal due to intracranial and intraabdominal hemorrhage. Therefore, early diagnosis is crucial to avoid life-threatening events.

The mechanisms of thrombocytopenia can be classified as follows: (1) pseudo-thrombocytopenia (an in vitro error); (2) decreased production (due to aplastic anemia, myelodysplastic syndrome, and nutritional disorders (vitamin B_12 _and folic acid deficiency)) or severe liver damage (decreased thrombopoietin production); (3) increased destruction and consumption (due to primary immune thrombocytopenic purpura (ITP), secondary ITP (associated with autoimmune diseases or *H. pylori* infection), Evans syndrome, thrombotic thrombocytopenic purpura (TTP), hemolytic uremic syndrome (HUS), and disseminated intravascular coagulation (DIC)); (4) dilution associated with massive rehydration or transfusion (excluding platelet transfusion); and (5) abnormal platelet distribution (splenic hyperplasia such as in portal hypertension) [4,5]. To differentiate between these mechanisms, it is necessary to obtain a wide range of information, including physical and laboratory findings and the patient’s previous and current medical history. The most important findings to be considered in blood tests are morphological abnormalities in blood cells and other hematologic findings [5]. For example, in TTP, HUS, and DIC, crushed erythrocytes and evidence of hemolytic anemia will be present. Blasts and nucleated erythrocytes will be seen in smears in bone marrow diseases. Thrombocytopenia without any other abnormal findings in the blood smear, as in this case, could indicate drug-related events, infection, splenic hyperfunction, primary ITP, or secondary ITP due to autoimmune diseases or *H. pylori* infection. In this case, there were no recently modified medications that could have caused thrombocytopenia, such as famotidine and heparin, which could potentially cause heparin-induced thrombocytopenia [5,6]. No signs of infection or findings suggestive of liver disease or splenic hyperfunction were observed. Hence, the most likely diagnosis in this case was ITP. ITP can be primary or secondary [7,8]. Primary ITP is a diagnosis of exclusion; it can be considered after all other thrombocytopenic diseases are ruled out. The typical causes of secondary ITP are SLE, antiphospholipid syndrome (APS), and *H. pylori *infection [7,8]. In this case, the patient had *H. pylori *IgG antibodies but was negative for stool antigens, which suggested a past infection. Platelet-associated IgG antibodies (PA-IgG) and anti-CD39 (platelet GP IIIb) autoantibodies induce immune thrombocytopenic purpura and lupus-associated thrombocytopenia [9]. In this case, we failed to measure PA-IgG before induction therapy, but it was elevated after high-dose glucocorticoid therapy. It suggested that PA-IgG could be responsible for thrombocytopenia cooperating with antiphospholipid (aPL) antibodies, although this inference might involve a risk of overestimation due to low specificity to the cause of thrombocytopenia [10]. The patient had aPL, which would suggest SLE and/or APS. Since she had no history of thrombosis or infertility, no current thrombotic events, and no multi-organ damage, primary or catastrophic APS was unlikely to be the underlying disease. In contrast, hemolytic anemia and aPL antibodies are likely to coexist in ITP with SLE [11,12]. Therefore, she was diagnosed with ITP secondary to SLE in the presence of aPL antibodies.

SLE causes damage to various organs throughout the body owing to autoantibody production and the deposition of immune complexes [1,6]. Thrombocytopenia is one of the typical manifestations, occurring in approximately 10-40% of patients with SLE [1]. It is included in the 1997 ACR classification and the 2012 Systemic Lupus International Collaborating Clinics classification; a platelet count <100,000/µL is considered to indicate thrombocytopenia in both criteria. Severe thrombocytopenia (≤50,000/μl) develops in 3-10% of patients with SLE [2,13]. The primary mechanism of thrombocytopenia in SLE is the production and binding of autoantibodies targeting GPIIb/IIIa, a platelet membrane protein that is recognized and phagocytosed by macrophages and subsequently trapped in the spleen [7]. SLE-induced thrombocytopenia is also associated with other pathological conditions, such as renal failure, hemolytic anemia, and central nervous system disorders. Although composite measures assessing lupus activity, such as the SLE disease activity index and the British Isles Lupus Assessment Group Index, include thrombocytopenia, the severity of decreased platelet count is not always reflected in the manifestations of lupus [14,15]. Severe thrombocytopenia is associated with an increased risk of bleeding complications and other fatalities [1]. Thrombocytopenia occurs in 20-50% of patients with primary APS and indicates a poor prognosis due to thrombosis during long-term observation [7]. However, some cases of thrombocytopenia with positive aPL antibodies in the absence of thrombotic events have also been reported [7,16,17]. Platelet counts in patients with ITP and positive aPL antibodies were 11,000 ± 11,000/µL. The presence of an aPL antibody shows no impact on the platelet counts in ITP [12]; therefore, severe thrombocytopenia in this case seemed relatively rare. Although the pathogenesis of aPL antibody-related thrombocytopenia is not clearly understood, several different mechanisms have been considered, including the binding of autoantibodies to GPIIb/IIIa or the anti-β_2_GPI-β_2_GPI complex to receptors on the platelet membrane. This results in the reduction or aggregation of platelets [7]. Moreover, aPL antibodies may mediate platelet destruction by binding to the phospholipids in the platelet membrane [17].

The treatment strategy for SLE varies according to the patient's manifestations and organ damage [18]. The initial therapy for thrombocytopenia is 1 mg/kg/day of prednisolone. Intravenous cyclophosphamide or methylprednisolone pulse therapy can be considered depending on the severity of symptoms. To maintain remission, prednisolone can be tapered with the concomitant introduction of HCQ, independent of the patient’s severity [6,18]. If patients are unresponsive or refractory to therapy, other immunosuppressive agents, such as azathioprine and tacrolimus, may be considered [6,18]. In ITP, rituximab and thrombopoietin receptor agonists are effective in patients refractory to prednisolone therapy. HCQ may be as effective as danazol and dapsone and has the potential for steroid-sparing effects in ITP treatment [19]. As TPO receptor agonists have no evidence of safety in SLE and may be a risk factor for thromboembolism, they have not been recommended for aPL-positive cases [18]. In lupus-related thrombocytopenia, complete remission (CR, defined as at least two cycles of ³100,000/µL platelet count) can be achieved in approximately 85% of patients with appropriate therapy. Furthermore, the mortality rate in patients with CR is significantly lower than that in those without CR [1]. In the present case, although the cause was unknown at the time of admission, an autoimmune mechanism for thrombocytopenia was suspected. Since the patient was recognized to be at an extremely high risk of bleeding, we decided to administer methylprednisolone pulse therapy. Concomitant use of HCQ and prednisolone improved the patient’s thrombopoiesis, leading to an increase in platelet count from 5,000 to 111,000/μl and a decrease in IPF from 14.9% to 2.6%. This result suggests that autoimmune-mediated platelet destruction can be successfully suppressed. CR was achieved in this case, indicating that the treatment was effective. In patients with SLE-induced severe thrombocytopenia, we recommend methylprednisolone pulse therapy and concomitant use of HCQ during prednisolone tapering to maximize efficacy and avoid corticosteroid-related side effects. In cases of resistance to high-dose glucocorticoid plus HCQ or intolerance to immunosuppressants, apheresis using a double filtration membrane may potentially improve refractory SLE-related thrombocytopenia associated with PA-IgG in addition to glucocorticoid therapy [20]. Further studies are required to determine appropriate therapeutic modalities for SLE-related severe thrombocytopenia.

## Conclusions

Here, we report a case of severe thrombocytopenic purpura in a patient with SLE. Although thrombocytopenia is a common complication of SLE, its severe form is relatively rare. This case provides important information regarding the efficient diagnosis and better treatment modalities for severe thrombocytopenia. Seemingly quiescent lupus without a history of thrombocytopenia can cause sudden severe thrombocytopenia, possibly in association with aPL antibodies, and high-dose glucocorticoid and HCQ administered at an appropriate time may induce a great recovery of thrombocytopenia and reduce the risk of life-threatening hemorrhage.
